# Successful Treatment of Pericardial Drainage and Platinum-Based Chemotherapy in a Case of BRCA-Positive Serous Peritoneal Carcinoma Complicated by Cardiac Tamponade

**DOI:** 10.7759/cureus.94150

**Published:** 2025-10-08

**Authors:** Akinori Sasaki, Rika Kimura

**Affiliations:** 1 Department of Oncology, Tokyo Bay Urayasu Ichikawa Medical Center, Urayasu, JPN

**Keywords:** brca1 mutation, carboplatin-paclitaxel and bevacizumab, cardiac tamponade, malignant pericardial effusion, peritoneal serous carcinoma

## Abstract

Cases of serous peritoneal carcinoma complicated by cardiac tamponade due to cancerous pericardial effusion are extremely rare and have poor prognoses. We report a case of a 72-year-old patient who presented with epigastric discomfort and was diagnosed with serous peritoneal carcinoma complicated by cardiac tamponade. The patient underwent emergency pericardial drainage for the cardiac tamponade. Subsequently, she was diagnosed with BRCA1-positive peritoneal carcinoma and was administered carboplatin, paclitaxel, and bevacizumab. After treatment initiation, cancer antigen 125 levels decreased rapidly, and no recurrence of pericardial effusion was observed. CT after four cycles of chemotherapy showed the disappearance of peritoneal dissemination and ascites. The patient completed six courses of induction chemotherapy and was scheduled to undergo maintenance therapy with PARP inhibitors. We report the first case of effective platinum-based chemotherapy in a patient with BRCA-positive peritoneal cancer complicated by cardiac tamponade. Chemotherapy has advanced dramatically in recent years, and even in cases with complications, such as cardiac tamponade, appropriate treatment can lead to a favorable long-term prognosis.

## Introduction

Ovarian and peritoneal carcinomas are estimated to affect >310,000 people and cause >200,000 deaths worldwide each year, with an increasing annual incidence rate [1]. Epithelial cancers arising from the ovary, fallopian tube, and peritoneum share similar clinical features and biological behavior and are therefore collectively referred to as epithelial ovarian cancer in both clinical practice and clinical trials. Most of these cancers are ovarian carcinomas, with peritoneal carcinoma accounting for approximately 10% of the total [2]. Ovarian and peritoneal carcinomas are often diagnosed at an advanced stage, with intraperitoneal metastases. In many cases, chemotherapy is necessary in addition to surgery. However, ovarian and peritoneal carcinomas are highly sensitive to platinum-based anticancer drugs. Therefore, a prognosis of approximately 30-40 months can be expected even in patients with stage IV cancer [3]. Pericardial effusion caused by ovarian and peritoneal carcinomas is extremely rare and has a poor prognosis [4]. In addition, in some cases of malignant pericardial effusion, significant accumulation of pericardial fluid causes circulatory dysfunction, leading to cardiac tamponade. Patients with cancer who develop cardiac tamponade require emergency pericardiocentesis and drainage; however, previous reports have indicated that these patients have a poor prognosis even when treated with cytotoxic chemotherapy [4,5].

In recent years, genetic testing for BRCA and homologous recombination deficiency (HRD) has become available for patients with ovarian and peritoneal carcinomas. HRD refers to a condition in which there is an abnormality in the homologous recombination repair pathway, the DNA repair mechanism. HRD positivity is observed in approximately 50% of patients with ovarian and peritoneal carcinomas. BRCA is a common cause of HRD and is found in approximately 20% of patients with ovarian and peritoneal carcinomas [6]. Approximately 70% of all BRCA mutations are germline, and about 30% are somatic [7]. Furthermore, patients with ovarian and peritoneal carcinomas positive for BRCA mutations are known to be more sensitive to platinum-based anticancer drugs (e.g., carboplatin) than those negative for BRCA mutations [8]. The reasons for this include that platinum-based anticancer drugs induce DNA inter- and intra-strand crosslinks, which ultimately result in double-strand breaks. In normal cells, damage can be repaired by homologous recombination repair. However, BRCA-deficient cells cannot repair the damage and undergo cell death, making them highly sensitive to platinum [9].

Herein, we report the case of a patient diagnosed with serous peritoneal carcinoma following cardiac tamponade. The patient harbored a BRCA1 mutation and responded to carboplatin-based chemotherapy. The patient provided informed consent for the presentation of clinical information in an anonymized form.

## Case presentation

**Figure 1:**
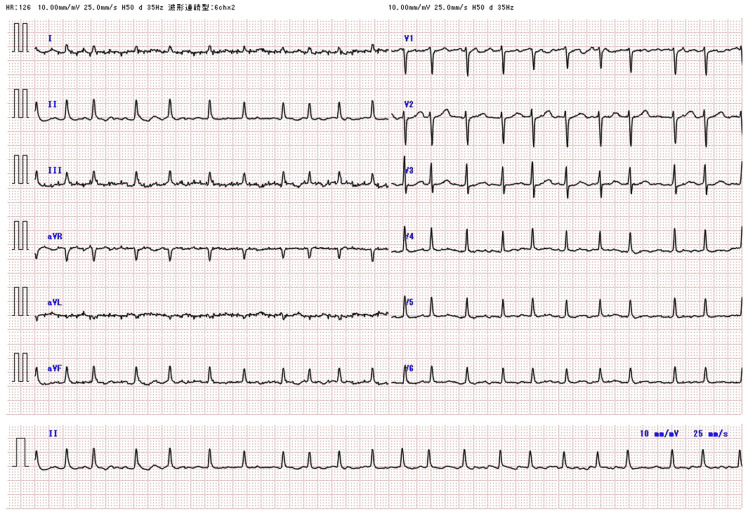
Electrocardiography in emergency departments The image reveals low QRS voltage in the limb leads during rapid atrial fibrillation.

**Figure 2:**
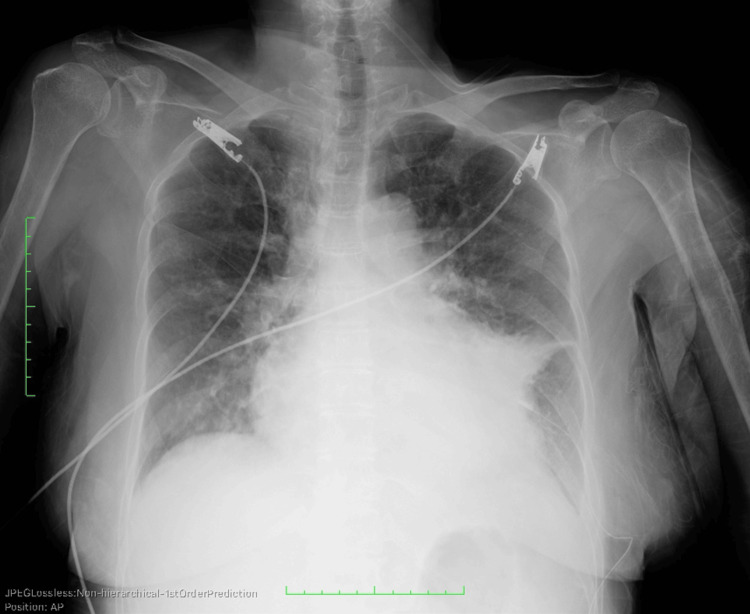
Chest radiography of the patient The image shows massive cardiomegaly and left pleural effusion.

**Figure 3:**
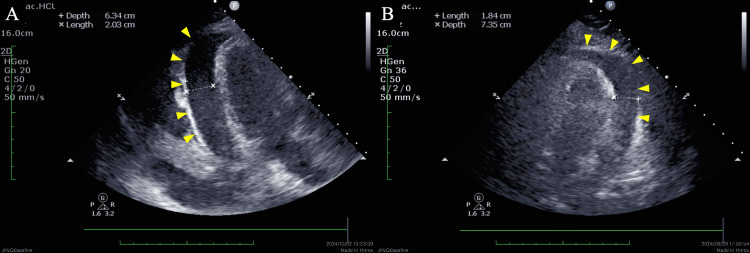
Echocardiography in emergency departments Echocardiography (A, B) shows massive pericardial effusion around the left ventricle (arrowheads).

**Figure 4:**
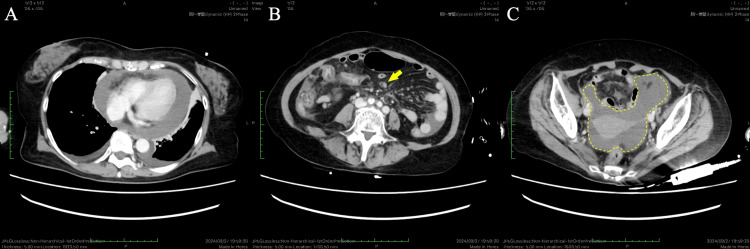
CT images at diagnosis Chest CT (A) reveals massive pericardial effusion and mild bilateral pleural effusion. Abdominal CT (B, C) reveals peritoneal dissemination (arrow) and accumulation of ascites in the pelvic cavity (dotted circle).

A 72-year-old female was referred to our hospital with a complaint of chest discomfort that had persisted for several days. The medical history included cervical spondylotic myelopathy. On physical examination in the emergency department, the patient’s blood pressure was 80/55 mmHg, and her pulse was 154 beats/min. Laboratory tests revealed elevated levels of cardiac enzymes (troponin I, 0.647 ng/mL; brain natriuretic peptide, 29.5 pg/mL). Electrocardiography revealed sinus tachycardia and a low voltage (Figure 1). Chest radiography revealed massive cardiomegaly and bilateral pleural effusion (Figure 2). Echocardiography demonstrated a large pericardial effusion accompanied by right ventricular diastolic collapse (Figure 3). Based on these findings, the patient was diagnosed with cardiac tamponade. An emergency pericardiocentesis via the subxiphoid approach yielded 300 mL of hemorrhagic effusion. Cytological analysis of the fluid confirmed malignant adenocarcinoma cells. Thus, the patient was diagnosed with cardiac tamponade due to malignant pericardial effusion. Chest and abdominal CT revealed peritoneal dissemination and moderate ascites; however, no obvious primary tumor was found (Figure 4). Upper and lower endoscopies were also performed; however, no primary tumors were found. Therefore, a cell block was prepared from the collected pericardial fluid, and immunohistochemical (IHC) staining was performed. IHC staining confirmed that Wilms tumor 1 protein showed nuclear positivity in the tumor cells. Additionally, IHC staining further indicated that the tumor cells were positive for cytokeratin 7 and the estrogen receptor and negative for cytokeratin 20. Laboratory tests revealed significantly elevated levels of cancer antigen 125 (CA125, 5408 U/mL), leading to the diagnosis of peritoneal carcinoma. The cell block was diagnosed as serous peritoneal carcinoma based on its histological type.

**Figure 5:**
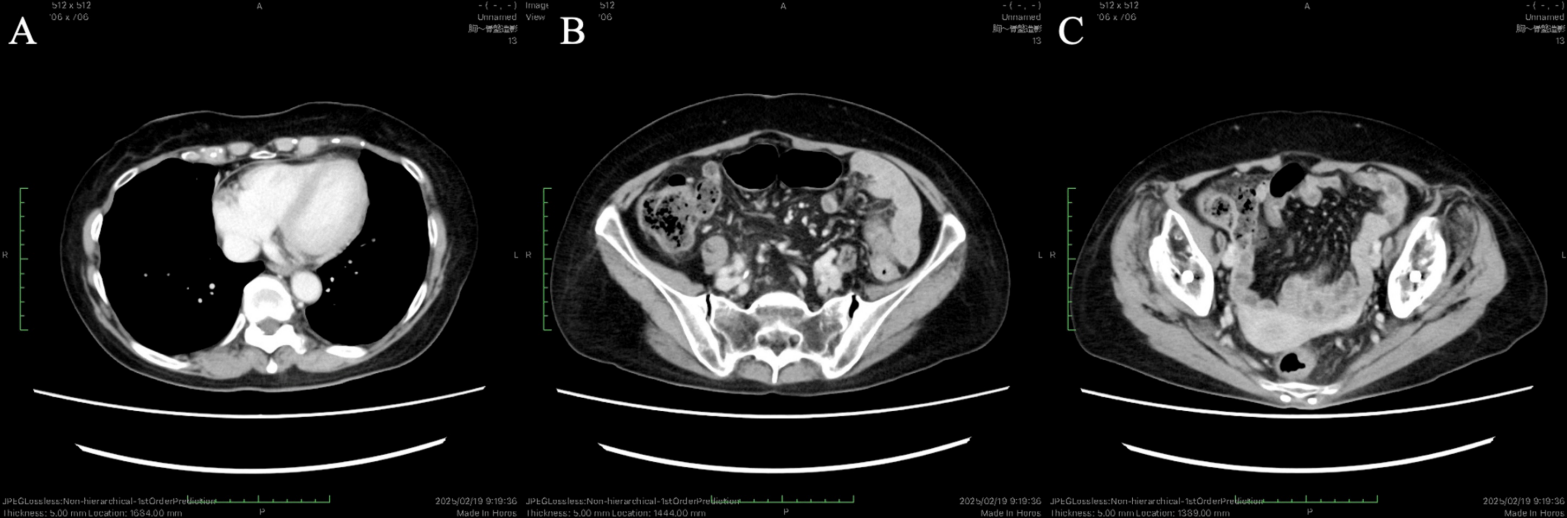
CT images obtained after six cycles of treatment with paclitaxel, carboplatin, and bevacizumab Follow-up chest and abdominal CT demonstrates complete resolution of the pericardial and pleural effusions, peritoneal dissemination, and ascites, corresponding to a complete response according to the RECIST criteria. RECIST, Response Evaluation Criteria in Solid Tumors

**Figure 6:**
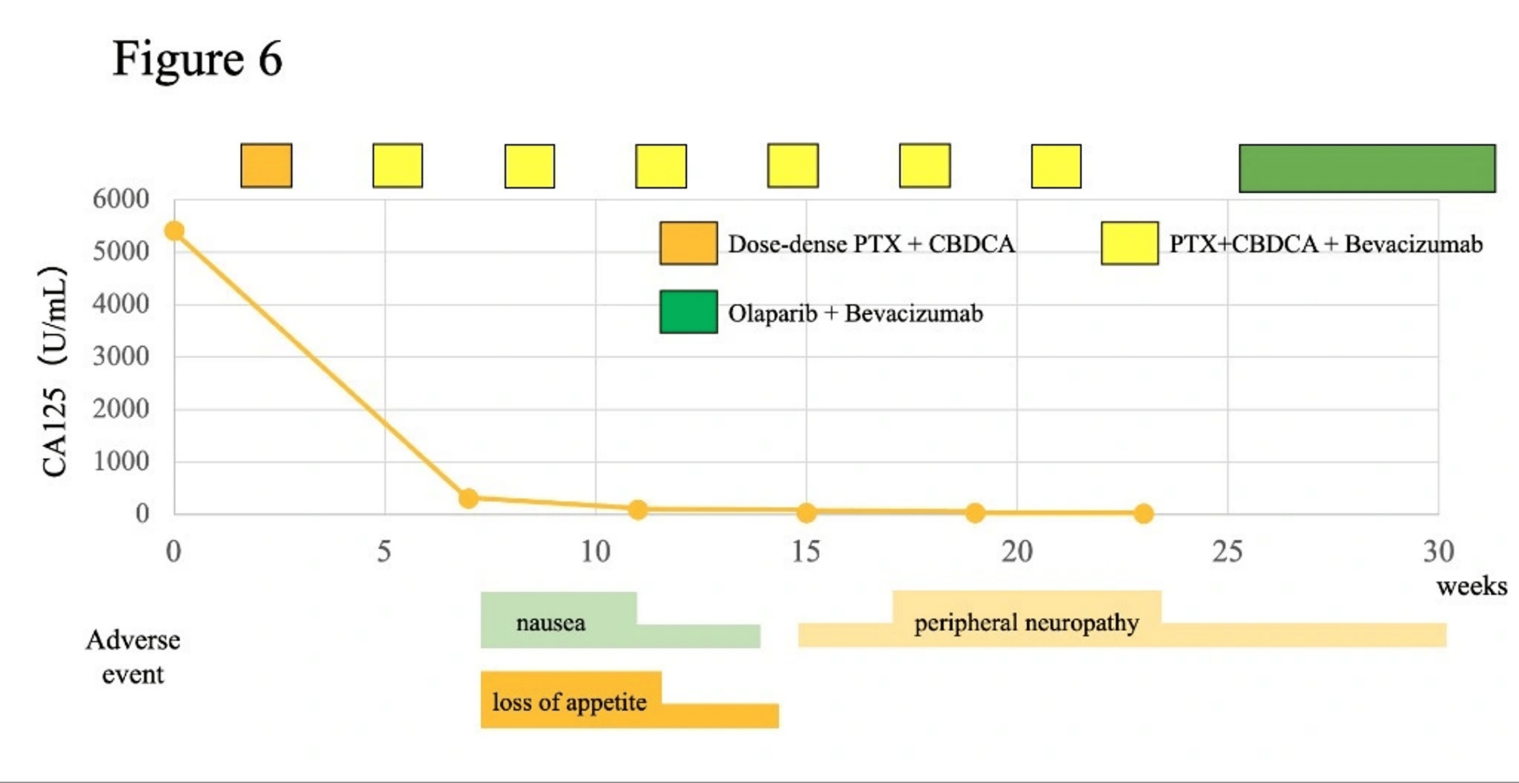
Course of treatment Course of tumor marker and adverse events while receiving chemotherapy.

The patient's symptoms disappeared, and her general condition improved after pericardial drainage; therefore, she was determined to be a candidate for chemotherapy, which was initiated two weeks after admission. The patient received initial chemotherapy with a dose-dense paclitaxel plus carboplatin regimen every three weeks: paclitaxel, 80 mg/m^2^ (days 1, 8, and 15); carboplatin area under the curve, 5 mg/mL×min (day 1). A BRCA1 mutation was revealed through germline BRCA analysis performed during the initial course on blood samples using the Sanger sequencing method. Based on this result, and in accordance with the findings of the PAOLA-1 trial, the treatment regimen was changed from the second course to paclitaxel, carboplatin, and bevacizumab [10]. The patient experienced grade 2 nausea and anorexia because of chemotherapy, which were tolerated by reducing the doses of paclitaxel and carboplatin. The CA125 level significantly decreased from 5408 U/mL at diagnosis to 120 U/mL after three cycles of chemotherapy. A CT scan at three months showed the disappearance of pericardial effusion, pleural effusion, peritoneal dissemination, and ascites, corresponding to a complete response (CR) according to the Response Evaluation Criteria in Solid Tumors (RECIST) version 1.1 (Figure 5). Interval debulking surgery was deemed unfeasible because of the risk of recurrent malignant pericardial effusion, the patient’s overall condition, and surgical risk. Therefore, the patient received maintenance therapy with olaparib and bevacizumab after paclitaxel, carboplatin, and bevacizumab therapy. Genetic counseling was recommended given the germline BRCA1 mutation, but the patient declined, as she had no siblings or children. Currently, the patient has been undergoing maintenance therapy for six months without tumor progression (Figure 6).

## Discussion

Here, we report a case of BRCA1 mutation-positive peritoneal carcinoma with cardiac tamponade due to malignant pericardial effusion that showed CR to platinum-based chemotherapy according to the RECIST criteria. Although BRCA mutation-positive peritoneal carcinoma is known to be highly sensitive to platinum, the effectiveness of platinum-based drugs against malignant pericardial effusion causing cardiac tamponade remains poorly documented. To our knowledge, this may be one of the first reported cases in this setting.

Common primary tumors involving the pericardium are lung and breast cancers. Other common tumors that metastasize to the heart include breast cancer, esophageal cancer, melanoma, and hematologic malignancies [11,12]. However, malignant involvement of the pericardium secondary to the metastatic spread of ovarian and peritoneal cancers is rare, accounting for only a small percentage of cases. This is because the primary modes of spread are retroperitoneal lymphatic dissemination and intraperitoneal surface dissemination in the ovarian and peritoneal cancers [13]. Although carcinomatous pericarditis may develop in association with disease progression in ovarian cancer, cases presenting with malignant pericardial effusion or cardiac tamponade at the initial diagnosis are extremely rare. The mechanism of malignant tumor metastasis to the pericardium has been reported as follows: First, cancer cells metastasize to the mediastinal lymph nodes, thereby blocking ascending mediastinal lymph flow. Second, cancer cells reach the pericardial cavity through the reverse flow of lymph [14]. Malignant pericardial effusion presents with nonspecific symptoms, such as dyspnea on exertion and rest, chest pain, cough, fatigue, and orthopnea [15]. Physical examination of cardiac tamponade may reveal Beck’s triad (tachycardia, hypotension, and muffled heart sounds), pulsus paradoxus, and the Kussmaul sign [16]. Chest radiography, electrocardiography, and echocardiography are useful for timely and accurate diagnosis of malignant pericardial effusion. Chest radiograph generally shows widening of the cardiac silhouette with clear lungs (the “water bottle” sign). Electrocardiogram sometimes shows low QRS voltage in the limb leads due to impaired transmission across the pericardial fluid, nonspecific ST- or T-wave changes, atrial ﬁbrillation, ventricular tachycardia, and complete atrioventricular block [15]. Echocardiography is the most useful diagnostic test for cardiac tamponade. Cardiac tamponade findings include pericardial effusion, right atrial systolic collapse, and right ventricular diastolic collapse on echocardiography [17]. Patients with pericardial effusion complicated by cardiac tamponade require urgent drainage. However, in many cases, pericardial fluid recidivates, making control of pericardial effusion difficult [18]. Perri et al. reported the prognosis of seven patients with ovarian cancer complicated by cardiac tamponade and concluded that the prognosis was poor. In the report, some patients died within three weeks, but one patient survived for up to 72 weeks [5].

Approximately 75% of patients with ovarian and peritoneal carcinomas present with stage III disease (characterized by peritoneal dissemination or lymph node involvement) or stage IV disease (defined by distant metastases) at diagnosis. The standard initial management for women with stage III or IV disease is primary surgical cytoreduction followed by systemic chemotherapy. However, neoadjuvant chemotherapy may be considered for patients who are poor surgical candidates due to the extent and distribution of disease or significant comorbidities at presentation. Combination therapy with carboplatin and paclitaxel is recommended as the first-line chemotherapy for ovarian and peritoneal carcinomas. Additionally, HRD-positive patients, including those with BRCA mutations, are highly sensitive to platinum drugs [8]. Therefore, higher treatment efficacy can be expected in these patients than in those without BRCA mutations or HRD. In a recent randomized phase III trial, PAOLA-1, initial treatment with carboplatin and paclitaxel plus bevacizumab, followed by maintenance therapy with olaparib plus bevacizumab, showed favorable results in patients with BRCA-positive ovarian carcinoma [10]. Based on this clinical trial, we selected paclitaxel, carboplatin, and bevacizumab therapy for our patient. The patient's tumor marker (CA125) level decreased significantly after the initiation of chemotherapy, and CT scans showed that ascites and peritoneal dissemination had disappeared. Moreover, her pericardial effusion significantly decreased, and no further drainage was required. Based on our experience, we believe that even in cases of ovarian and peritoneal carcinomas complicated by cardiac tamponade with a poor prognosis, long-term prognosis can be expected if appropriate examinations and treatments are administered to these patients.

## Conclusions

In conclusion, to the best of our knowledge, this may be one of the first reported cases of an exceptional response to platinum-based chemotherapy in a patient with BRCA1-positive peritoneal carcinoma complicated by cardiac tamponade. Cardiac tamponade caused by cancerous pericardial effusion significantly worsens the prognosis, and there are some cases in which chemotherapy is not administered and only palliative care is provided. In contrast, there are cases similar to ours in which chemotherapy is highly effective, allowing patients to resume their daily lives. In recent years, various chemotherapy regimens have been approved, and the prognosis of patients with cancer has improved dramatically. Therefore, cases in which chemotherapy has become possible are now being reported, particularly in patients with a poor prognosis who were previously not considered candidates for chemotherapy. Even patients with cancer who develop serious complications can expect an improved prognosis through appropriate testing and treatment. However, as this is a report of a single case, caution is warranted in generalizing the potential for long-term prognosis, and further studies with larger cohorts and longer follow-up are needed.
